# Cardioprotective activity of iron oxide nanoparticles

**DOI:** 10.1038/srep08579

**Published:** 2015-02-26

**Authors:** Fei Xiong, Hao Wang, Yidong Feng, Yunman Li, Xiaoqing Hua, Xingyun Pang, Song Zhang, Lina Song, Yu Zhang, Ning Gu

**Affiliations:** 1 State Key Laboratory of Bioelectronics, Jiangsu Laboratory for Biomaterials and Devices, School of Biological Science and Medical Engineering, Southeast University, 2 Sipailou, Nanjing 210096, China; 2 State of Key laboratory of Natural Medicines, China Pharmaceutical University, 24 Tongjiaxiang, Nanjing 210009, China

## Abstract

Iron oxide nanoparticles (IONPs) are chemically inert materials and have been mainly used for imaging applications and drug deliveries. However, the possibility whether they can be used as therapeutic drugs themselves has not yet been explored. We reported here that Fe_2_O_3_ nanoparticles (NPs) can protect hearts from ischemic damage at the animal, tissue and cell level. The cardioprotective activity of Fe_2_O_3_ NPs requires the integrity of nanoparticles and is not dependent upon their surface charges and molecules that were integrated into nanoparticles. Also, Fe_2_O_3_ NPs showed no significant toxicity towards normal cardiomyocytes, indicative of their potential to treat cardiovascular diseases.

Cardiovascular disease is the leading cause of death worldwide and the deaths have increased at a fast rate^1^. Myocardial ischemia, caused by lack of blood flow to the heart, is the most common and primary cause of myocardium damage. This, in turn, leads to myocardial hypoxia. Myocardial ischemia and hypoxia can cause coronary artery heart disease, angina pectoris, even myocardial infarction. Treatment for myocardial ischemic injury is timely and effective myocardial reperfusion for improving blood flow to the heart muscle. However, the process of reperfusion can in itself paradoxically inflict further injury to the myocardium, including cardiomyocyte death, arrhythmia^2^, even cardiac rupture^3^. A variety of pharmaceutical drugs have been investigated, including oxygen free radical scavengers, antioxidants, calcium channel blockers, anti-apoptotic agents and so forth. Myocardial injury is associated with multiple pathological mechanisms, while a pharmacologically active compound fights only one of them. Therefore there is still no proven effective therapy^3^.

IONPs, including Fe_2_O_3_ and Fe_3_O_4_ NPs, have been extensively used as medical diagnostic agents^4^, drug carriers^5^, hyperthermia for cancer treatment^6^, separation tools^7^, and cancer diagnoses and therapies. IONPs are generally considered as inert materials. Thus, to endow these NPs with biological properties, they are often bound to certain biologically active molecules such as antibodies, drugs, and DNAs to create nano-composites. Interestingly, it has been recently reported that Fe_3_O_4_ NPs in a catalytic reaction *in vitro* show a peroxidase-like activity in a manner depending upon sizes in a range from 30 to 300 nm^8^. After that, a dual enzyme (peroxidase and catalase-like) activities of IONPs *in vitro* were reported and the relative potency of Fe_3_O_4_ NPs is higher than that of Fe_2_O_3_ NPs^9^. However, it is unclear whether IONPs themselves can actually be used as a drug to treat diseases. Here, we reported that 2, 3-dimercaptosuccinic acid modified Fe_2_O_3_ NPs (Fe_2_O_3_@DMSA NPs) in a range of small sizes exhibit a cardioprotective activity *in vitro* and *in vivo*. We also compared the activity with those of Verapamil (calcium channel blockers) and *Salvia miltiorrhiza* extract (antioxidant), two drugs that have been extensively used as cardioprotective drugs in the treatment of angina pectoris, coronary artery heart disease and so on^10^,^11^,^12^. Our data suggest that these NPs have a clinically potential to treat cardiovascular diseases.

## Results

### Cardioprotection of Fe_2_O_3_@DMSA NPs

**Figure 1:**
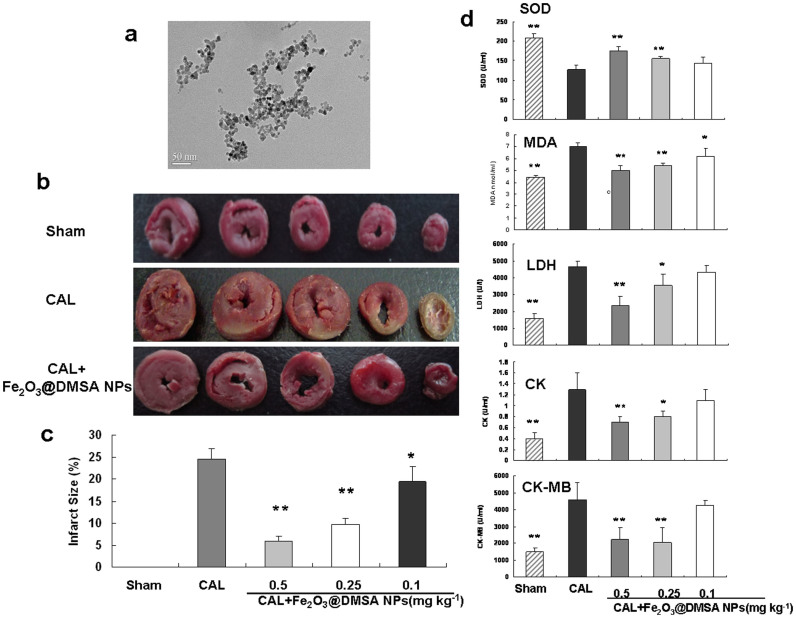
Fe_2_O_3_@DMSA NPs protected coronary artery ligature (CAL) induced injury in rats. (a) TEM image of Fe_2_O_3_@DMSA NPs of 9.8 nm. (b) Representative images for heart sections (stained with triphenyltetrazolium chloride solution) derived from the sham-operated control (Sham), normal saline-treated (CAL) and Fe_2_O_3_@DMSA NPs-treated (CAL + Fe_2_O_3_@DMSA NPs) rats at 0.25 mg kg^−1^. (c) Quantification of the size of heart infarcts of sham-operated control, normal saline- and Fe_2_O_3_@DMSA NPs-treated rats. (d) Quantification of several serum index (SOD, MDA, LDH, CK, and CK-MB) of sham-operated control, normal saline- and Fe_2_O_3_@DMSA NPs-treated rats. * *p* < 0.05 vs the CAL group; ** *p* < 0.01 vs the CAL group; *n* = 8.

We prepared Fe_2_O_3_@DMSA NPs by a co-precipitation method. The prepared NPs have a spherical core with an average diameter of 9.8 nm as measured by TEM (Fig. 1a). To analyze the potential ability of NPs to protect cardiac, the effect of NPs on the size of myocardial infarct and biochemical indexes were investigated at animal level by using a rat coronary artery ligature (CAL) model. The Sprague-Dawley rats were injected with Fe_2_O_3_@DMSA NPs (CAL + Fe_2_O_3_@DMSA NPs group, 0.1, 0.25, 0.5 mg Fe kg^−1^) or normal saline solution (CAL group) via tail veins once-a-day before induction of injury by CAL surgery. Fe_2_O_3_@DMSA NPs-treated rats had infarct regions significantly smaller than did the normal saline-treated group after 30 min of injury (Fig. 1b). The Fe_2_O_3_@DMSA NPs-mediated improvement was also dose-dependent with a highest improvement at 0.5 mg kg^−1^. To verify the protection, we measured several biochemcial indexes in serum, including the levels of superoxide dismutase (SOD), malondialdehyde (MDA), lactate dehydrogenase (LDH), creatine kinase (CK) and creatine kinase isoenzyme-MB (CK-MB). The SOD activity of the Fe_2_O_3_@DMSA NPs-treated rats at 0.5 and 0.25 mg kg^−1^ doses was significantly higher than that of the normal saline-treated rats. The activities of MDA, LDH, CK, and CK-MB and the content of MDA in Fe_2_O_3_@DMSA NPs-treated rats at the same doses were also significantly lower than those of the normal saline-treated rats. These results demonstrated that Fe_2_O_3_@DMSA NPs protected myocardium from ischemia injury at animal level.

**Figure 2:**
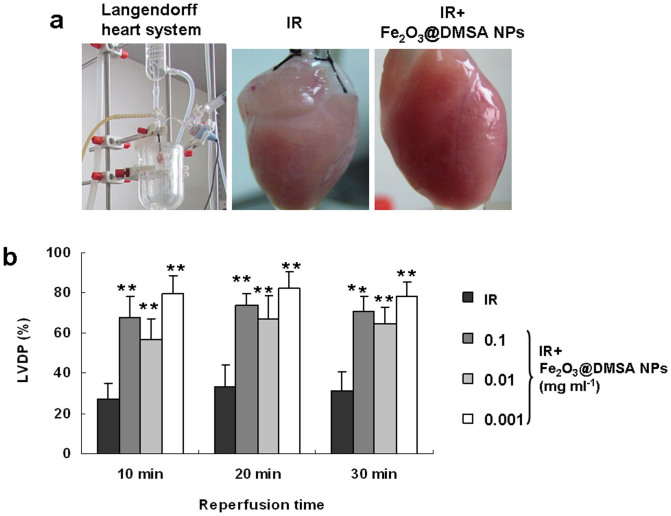
Fe_2_O_3_@DMSA NPs have a caridoprotective activity in guinea pig Langendorff heart following 30 min of ischemia and 30 min of reperfusion (IR). (a) The Langendorff heart system (left), a heart perfused without (IR, middle) and with 0.001 mg ml^−1^ of Fe_2_O_3_@DMSA NPs (IR + Fe_2_O_3_@DMSA NPs, right) after ischemia. (b) LVDPs were recorded with perfused hearts without and with 0.1, 0.01 and 0.001 mg ml^−1^ of Fe_2_O_3_@DMSA NPs at 10, 20 and 30 min after perfusion. ** *p* < 0.01 vs the IR group; *n* = 6.

To confirm the cardioprotective activity of Fe_2_O_3_@DMSA NPs, we prepared Guinea pig Langendorff heart (Fig. 2a), a widely used model at tissue level for assessing potential cardiovascular drugs. The perfused hearts were subjected to ischemia and reperfusion (IR) with or without Fe_2_O_3_@DMSA NPs. The treatment with Fe_2_O_3_@DMSA NPs did not change the heart rate significantly during reperfusion. However, compared with the untreated hearts (IR group), the hearts treated with Fe_2_O_3_@DMSA NPs (IR + Fe_2_O_3_@DMSA group) at 0.001–0.1 mg ml^−1^ within 30 min of reperfusion exhibited a significant recovery in the left ventricular developed pressure (LVDP) (Fig. 2b). Also, while the untreated hearts looked pale, the Fe_2_O_3_@DMSA NPs-treated hearts were ruddy and seemed to be normal in color (Fig. 2a middle and right).

**Figure 3:**
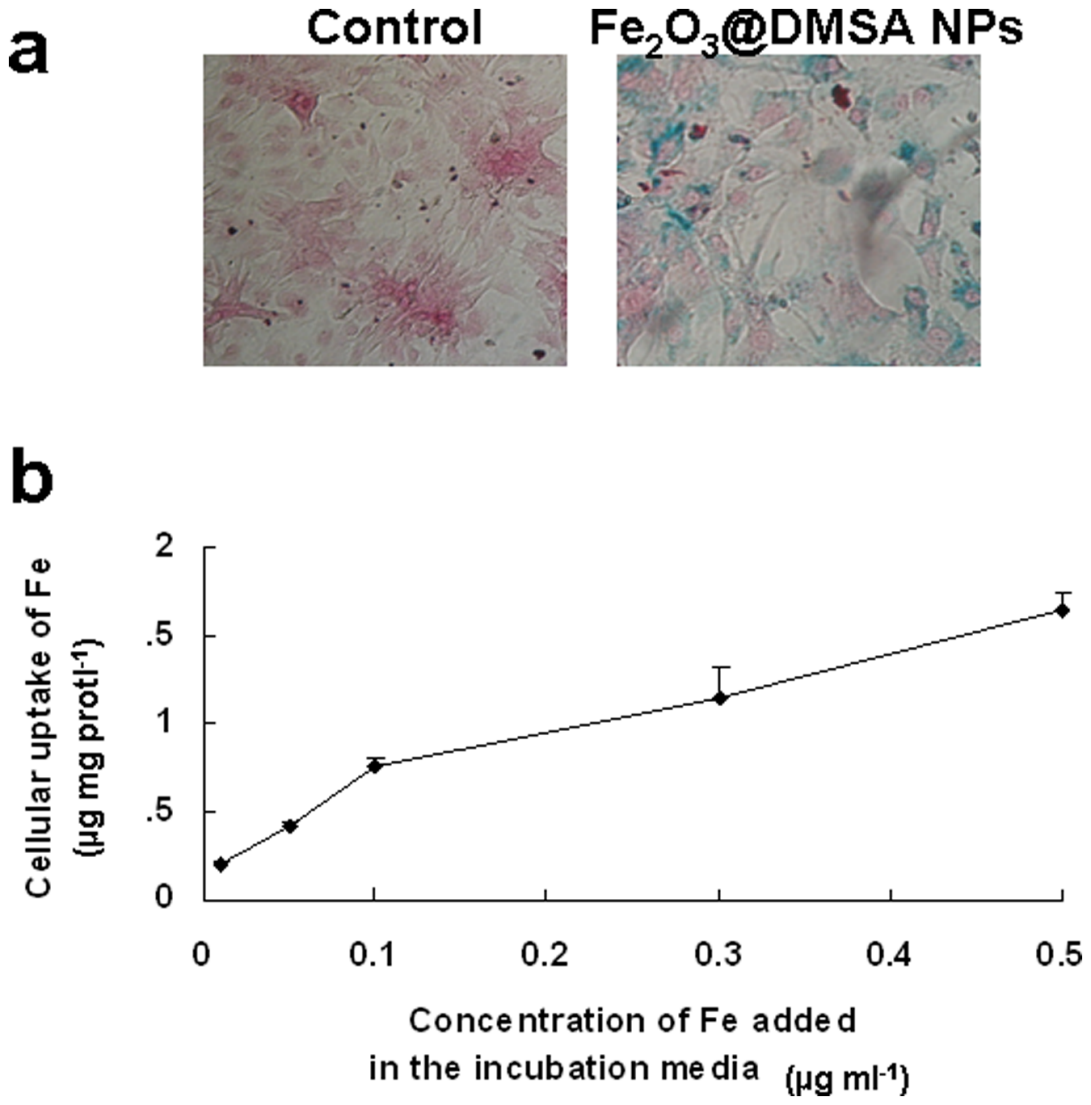
The uptake of Fe_2_O_3_@DMSA NPs by cardiomyocytes. (a) Prussian blue staining of cardiomyocytes incubated without (control) and with 0.1 mg ml^−1^ of Fe_2_O_3_@DMSA NPs. (b) The uptake of Fe_2_O_3_@DMSA NPs at different doses (*n* = 6).

To further understand the Fe_2_O_3_@DMSA NPs-induced cardioprotective activity, we investigated the effect of Fe_2_O_3_@DMSA NPs on neonatal rat cardiomyocytes. Cardiomyocytes were cultured without (control group) and with of NPs for 24 h, and cellular Fe_2_O_3_@DMSA NPs were visualized by Prussian blue staining (Fig. 3a). The uptake of NPs was apparently dose-dependent as detected by potassium ferrocyanide colorimetric assay (Fig. 3b). The viability of cardiomyocytes incubated with 0.01, 0.1 and 0.5 mg ml^−1^ of NPs was 94.0 ± 9.2, 94.6 ± 9.2 and 96.2 ± 7.1%, respectively. No significant difference (*p* > 0.05) of cell viability between 0 and 0.01–0.5 mg ml^−1^ of NPs was detected, indicating that the NPs at those doses were non-toxic to normal cardiomyocytes.

**Figure 4:**
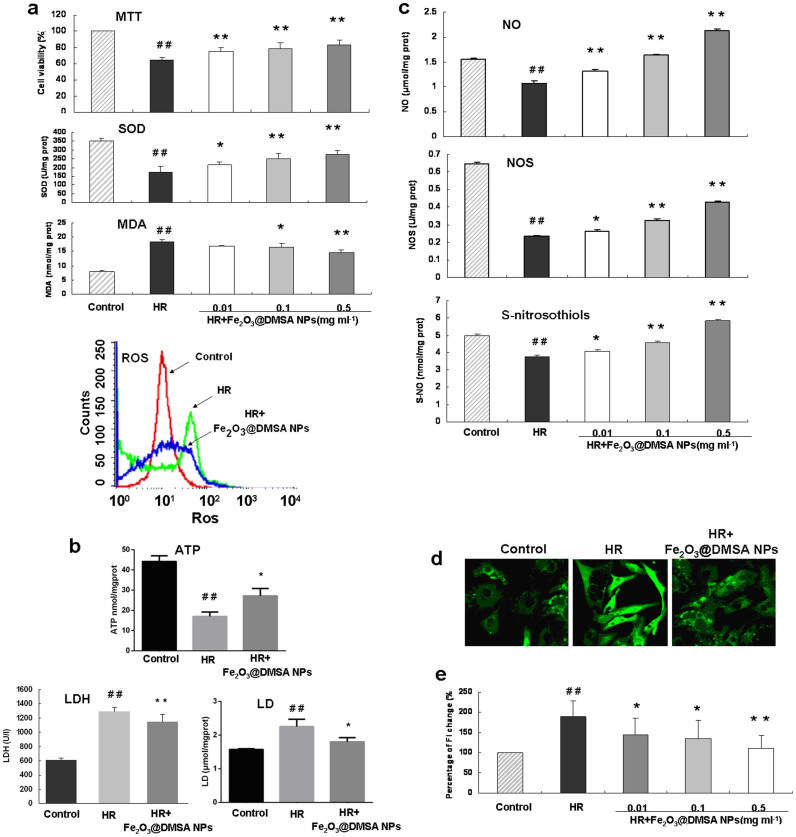
Effects of Fe_2_O_3_@DMSA NPs on cardiomyocytes under a hypoxia and reoxygenation (HR) condition. (a), Cardiomyocytes were exposed to hypoxia (95% N_2_ and 5% CO_2_) for 6 h followed by 6 h of reoxygenation (95% O_2_ and 5% CO_2_) (HR) and effects of Fe_2_O_3_@DMSA NPs on cell viability, generation of SOD, MDA and ROS (a), on LDH, LD and ATP (b), and on generation of NO, NOS and S-NO were measured (c). Confocal images of intracellular calcium signal of cardiomyocytes of control, HR and HR + Fe_2_O_3_@DMSA NPs (0.1 mg ml^−1^) group (d). The fluorescent intensity (FI), which indicates intracellular Ca2^+^ concentration, was quantified (e). * *p* < 0.05 vs the IR group; ** *p* < 0.01 vs the IR group; ^##^ *p* < 0.01 vs the control group; *n* = 6.

Hypoxia and reoxygenation (HR) is a cell model of heart ischemia and reperfusion injury. When cardiomyocytes were exposed to HR, Fe_2_O_3_@DMSA NPs treatment significantly increased the viability and cellular SOD activity, and decreased the content of cellular MDA and reactive oxygen species (ROS) (Fig. 4a). In addition, Fe_2_O_3_@DMSA NPs treatments significantly impacted on the cell energy metabolism as indicated by decrease of the generation of LDH and lactate (LD), and increase of the generation of adenosine triphosphate (ATP) (Fig. 4b). The contents of nitric oxide (NO), activity of nitric oxide synthase (NOS) and the level of S-nitrosothiols in cardiomyocytes were also significantly increased upon Fe_2_O_3_@DMSA NPs after HR (Fig. 4c). On the other hand, Fe_2_O_3_@DMSA NPs treatment significantly decreased intracellular calcium concentration in a dose-dependent manner (Fig. 4d and 4e). These results demonstrate Fe_2_O_3_@DMSA NPs could protect cardiomyocytes from HR injury in many aspects at cell level.

### Fe_2_O_3_ is critical for the activity of NPs

**Figure 5:**
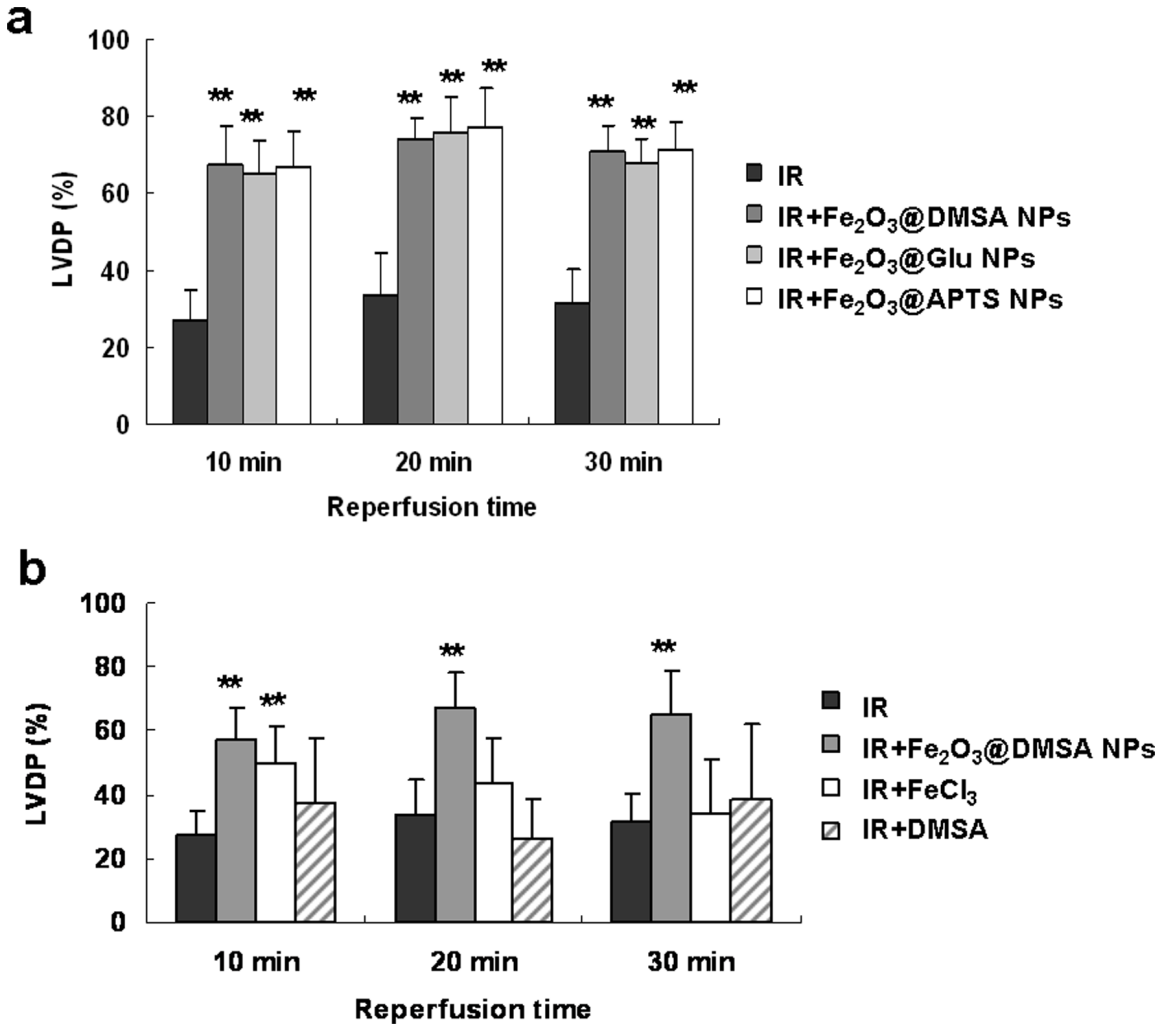
Charges, surface molecules and iron ions do not contribute to the cardioprotective activity in guinea pig Langendorff heart following IR. (a) Fe_2_O_3_ NPs were coated with three different molecules and used to treat perfused hearts. (b) Perfused hearts were treated with Fe_2_O_3_@DMSA NPs, FeCl_3_ solution and DMSA, respectively, and subjected to LVDP recovery examination. ** *p* < 0.01 vs the IR group, *n* = 6.

It is known that the surface charge and chemical components play an important role in many biological activities of NPs^13^,^14^,^15^. Therefore, it is possible that the negatively charged Fe_2_O_3_@DMSA NPs and surface factors might have attributed to the observed cardioprotective activity. To examine the possible role of charges, we prepared 3-amino-propyltriethoxysilane-modified NPs (Fe_2_O_3_@APTs NPs, which are positively charged) and L-glutamic acid-modified Fe_2_O_3_ NPs (Fe_2_O_3_@Glu NPs, which are near-neutrally charged) (Characterization are shown in Supplementary Fig. S1 and S2). The zeta potentials of Fe_2_O_3_@DMSA NPs, Fe_2_O_3_@APTs NPs and Fe_2_O_3_@Glu NPs were −43.1, 28.9 and −2.1 mV at pH 7.4, respectively. In IR model, however, all types of NPs with same concentration (0.1 mg ml^−1^) showed a significant potency in protecting perfused Langendorff hearts (Fig. 5a, *p* < 0.01 vs the IR group) and the difference between them was not significant (*p* > 0.05). Thus, the cardioprotective activity of the NPs does not depend on the surface charge.

To examine the possible role of surface molecules, the Langendorff hearts were perfused with 0.8 μg ml^−1^ DMSA, the concentration equivalent to that on the surface of 0.01 mg ml^−1^ Fe_2_O_3_@DMSA NPs. Treatment with DMSA alone did not impact significantly on the LVDP of Langendorff hearts (Fig. 5b, *p* > 0.05 vs the IR group), indicating that DMSA did not attribute to the observed cardioprotective activity of Fe_2_O_3_ NPs.

Next, we analyzed whether or not the cardioprotective activity was due to a trace amount of iron ions leached from Fe_2_O_3_@DMSA NPs. Thus, we measured the concentration of iron ions by ultrafiltrating 0.1 mg ml^−1^ Fe_2_O_3_@DMSA NPs in perfusion solution. However, we did not detect iron ions in the ultrafiltrate as measured by inductively coupled plasma atomic emission spectroscopy. Also, the ultrafiltrate had no effects on perfused hearts, indicating that the observed cardioprotective activity was likely due to intact NPs. To further confirm the necessity of NPs, Langendorff hearts were perfused with FeCl_3_ at the same iron concentration as that present in 0.01 mg ml^−1^ Fe_2_O_3_@DMSA NPs. Fig. 5c shows that the LVDP of FeCl_3_ solution-treated hearts was only transiently recovered at 10 min of reperfusion (*p* < 0.01 vs the IR group), while the activity of Fe_2_O_3_@DMSA NPs was more sustainable from 10 to 30 min. These results demonstrate that the integrity of NPs is essential for its optimal activity.

### The activity of Fe_2_O_3_@DMSA NPs with a smaller size is more effective than those with larger size

**Figure 6:**
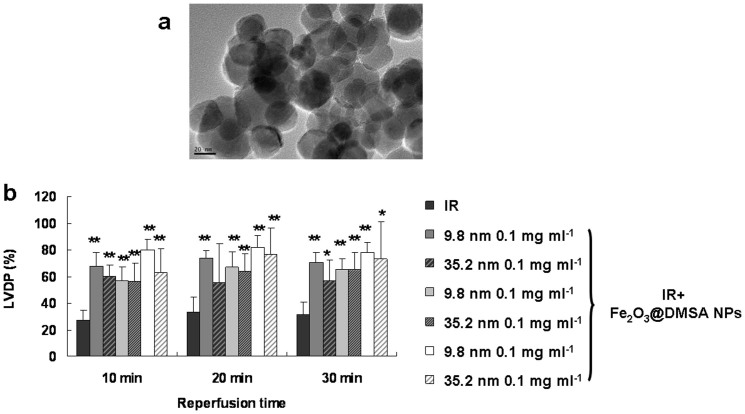
The cardioprotective activity of Fe_2_O_3_@DMSA NPs is dependent on their core sizes in guinea pig Langendorff heart following IR. (a) TEM image of Fe_2_O_3_@DMSA NPs of 35.2 nm. (b) Injured hearts were treated with Fe_2_O_3_@DMSA NPs of different sizes and then examined for LVDP recovery. * *p* < 0.05 vs the IR group; ** *p* < 0.01 vs the IR group; *n* = 6.

IONPs with different sizes often show different properties^16^,^17^. Thus, we studied the cardioprotective activities of Fe_2_O_3_@DMSA NPs of an average core size of 9.8 and 35.2 nm (Fig. 4a), respectively. Although Fe_2_O_3_@DMSA NPs of 35.2 nm recovered injured hearts significantly as indicated by improving LVDP (*p* < 0.01, vs the IR group), they were apparently less effective than Fe_2_O_3_@DMSA NPs of 9.8 nm in the doses ranging from 0.001 to 0.1 mg ml^−1^ (Fig. 6).

### Fe_2_O_3_@DMSA NPs protected heart injury more effectively than Verapamil and *Salvia miltiorrhiza* extract

**Figure 7:**
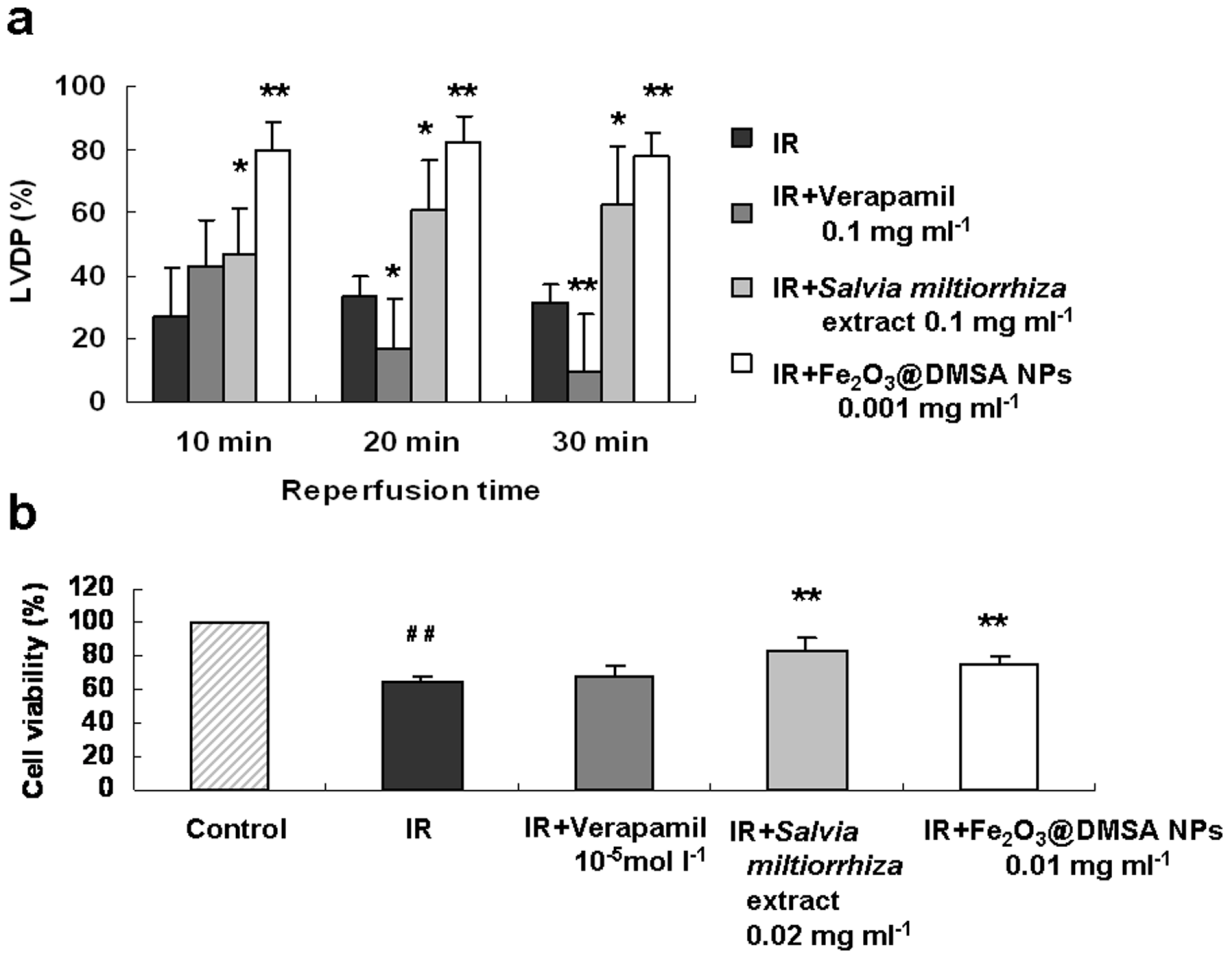
Comparison of cardioprotective effects of Fe_2_O_3_@DMSA NPs with Verapamil and *Salvia miltiorrhiza* extract. (a) LVDPs of guinea pig Langendorff hearts without (IR) and with 0.1 mg ml^−1^ of Verapamil (IR + Verapamil), 0.2 mg ml^−1^ of *Salvia miltiorrhiza* extract (IR + *Salvia miltiorrhiza* extract) and 0.001 mg ml^−1^ of Fe_2_O_3_@DMSA NPs (IR + Fe_2_O_3_@DMSA NPs) under IR condition. (b) Effects of Verapamil, *Salvia miltiorrhiza* extract and Fe_2_O_3_@DMSA NPs on the viability of cardiomyocytes under HR condition. * *p* < 0.05 vs the IR or HR group; ** *p* < 0.01 vs the IR or HR group; ^##^ *p* < 0.01 vs the control group; *n* = 6.

Certain natural organic molecules have been used in clinic for cardiovascular therapies. We compared Fe_2_O_3_@DMSA NPs with Verapamil (synthetic drug) and *Salvia miltiorrhiza* extract (natural product). Verapamil was reported to reduce ischemic injury of heart by inhibiting calcium influx^18^, just like Fe_2_O_3_@DMSA NPs, but it can not improve the LVDP recovery in guinea pig Langendorff heart under the condition where Fe_2_O_3_@DMSA NPs worked effectively (Fig. 7a). *Salvia miltiorrhiza* extract also improved injured hearts significantly (p < 0.05) at 0.1 mg ml^−1^. However, it was apparently less potent than Fe_2_O_3_@DMSA NPs at 10 min of reperfusion (p < 0.05). Also, unlike Fe_2_O_3_@DMSA NPs-treated rats, the hearts derived from Verapamil or *Salvia miltiorrhiza* extract–treated rats had a pale appearance as that of the IR group (Supplementary Fig. S3).

It has been reported that Verapamil is toxic to normal neonatal rat cardiomyocytes^19^,^20^ and may impact negatively on the function of thyroid and saccharide metabolism. Under HR, Verapamil also failed to improve the viability of injured cardiomyocytes (Fig. 7b), which is constant with previously reported results^21^. *Salvia miltiorrhiza* extract improved the viability of injured cells similarly as Fe_2_O_3_@DMSA NPs at a concentration 20-fold higher than that of Fe_2_O_3_@DMSA NPs.

## Discussion

Cardiovascular diseases are the leading cause of mortality in the worldwide^22^ and a variety of pharmaceutical drugs have been used to target specifically pathological processes for myocardial injury. Yet, these compounds are often associated with side effects and currently there is still no effective therapy to prevent heart injuries^23^. In this study we reported for the first time a cardioprotective activity of Fe_2_O_3_@DMSA NPs. Our results suggest that the NPs can protect heart from ischemic damages *in vivo* as well as *in vitro*. The cardioprotective activity of Fe_2_O_3_@DMSA NPs is dependent on the core sizes but independent on the molecules used to coat NPs. In addition, the integrity of “nanoparticle” is necessary for its protective activity.

The mechanism of IR injury is complex, including production of free radicals, calcium overload, altered energy metabolism etc. When the heart is exposed to IR, on one hand, ATP catabolism leads to adenosine diphosphate (ADP), adenosine monophosphate (AMP), adenine nucleoside, hypoxanthine nucleoside and then hypoxanthine. On the other hand, ATP depletion leads to the loss of calcium pump function and then the rise in membrane permeability to calcium. Increased concentration of intracellular calcium could activate the calmodulin-dependent protein kinase (CaM kinase),which could induce the conversion of xanthine dehydrogenase (XDH) to xanthine oxidase (XO)^18^. XO catalyzes hypoxanthine to produce xanthine and large amount of ROS (a kind of free radical)^19^. Fe_2_O_3_@DMSA NPs could inhibit intracellular ROS and then decrease the peroxidation injury of membrane lipid. MDA, the end-products of membrane lipid peroxidation, can also form a conjugated Schiff base product by reacting with the amino group of proteins or phospholipid of membrane^20^, which induce low fluidity, high permeability and damage of membrane^14^. Decrease of MDA could reduce membrane damage-induced intracellular LDH leakage to culture supernatant^19^ and extracellular Ca^2+^ entrance into cell along the concentration gradient^20^. Fe_2_O_3_@DMSA NPs could inhibit calcium influx, which could then inhibit the ROS. Recently, NO, synthesized from L-arginine by a group of hemoproteins (NOS), is recognized as an important mediator of pathological processes of IR injury^24^. Fe_2_O_3_@DMSA NPs could increase the NOS activity and then increase NO production. NO signal pathway also could be activated by decrease of XO-generated ROS^25^. Protein S-nitrosylation is a new type of protein post-translational modification, which is a reversible attachment of NO moiety to specific cysteine residues of selected proteins and then produces labile S-nitrosothiol structure and functional alternations. This modification could confer protective effects against myocardial IR^26^,^27^,^28^. Fe_2_O_3_@DMSA NPs could increase the level of S-nitrosothiols and then participate in NO-mediated protection against IR injury. In addition, S-nitrosation could control calcium channel and then inhibit calcium influx^29^. The molecular mechanisms by which ROS, NO and calcium modulate cellular signal transduction remain incompletely understood and crosstalk among ROS-, NO- and calcium-regulated pathways may occur under the Fe_2_O_3_@DMSA NPs treatment.

The cardioprotective activity of Fe_2_O_3_@DMSA NPs appeared to be higher than those of *Salvia miltiorrhiza* extract and Verapamil because the NPs showed equal or better protections at lower concentrations. Fe_2_O_3_@DMSA NPs also show a restorative effect on ischemic hearts and cell damages, the activity that was not observed with Verapamil. Our observed bioactivity of Fe_2_O_3_@DMSA NPs is apparently conflicted with a previously reports that IONPs have a peroxidative activity^8^,^30^ and are toxic to tumor cells^31^. The differential activity of IONPs may be due to (1) cell types, (2) experimental conditions (such as concentrations, under HR, and others), (3) the distribution and intracellular microenvironment of NPs, and (4) different composites of NPs. To explore the natural and basic laws of IONPs function require further study of the interaction between IONPs and more cell types under different physiological and pathological conditions.

Our discovered cardioprotective activity of Fe_2_O_3_@DMSA NPs may have a clinical implication because the NPs are easier to be prepared under the condition avoiding environmental contaminations. We expect that application of Fe_2_O_3_@DMSA NPs to treat cardiovascular diseases would reduce significantly the costs associated with currently used cardioprotective drugs that are made from either natural sources or chemical syntheses.

## Methods

### Synthesis and characterization of IONPs

Fe_2_O_3_ NPs with approximately 10 nm were synthesized and coated with 2, 3-dimercaptosuccinic acid (DMSA), 3-amino-propyltriethoxysilane (APTs) and L-glutamic acid (Glu) according to our previous work^4^,^32^. 35.2 nm Fe_2_O_3_ NPs were purchased from XCNM Co., Ltd (China) and coated with DMSA as the same method mentioned above. The core diameters of NPs were characterized by TEM (JEM-200CX, JEOL). The hydrodynamic diameters and zeta potential were measured by electrophoresis instrument (Brookhaven Zetaplus, Malvern).

### Coronary artery ligature and assessments

The animals used for the experiment were treated according to the protocols evaluated and approved by the ethical committee of Southeast University (Nanjing, China). Sprague-Dawley rats (200–250 g) were administrated with Fe_2_O_3_@DMSA NPs through tail veins. After 7 days, the treated rats were subjected to coronary artery ligature as described below. For the sham and the CAL group, rats were administrated with normal saline solution only.

To perform coronary artery ligature, rats were anesthetized through intraperitoneal injection with 10% chloral hydrate and subsequently fixed on an operation table in a supine position. The limbs of an anesthetized rat were connected to an electrocar diographer (ECG-6511, Nihon Kohden), which recorded the electrocardiogram of the animal's heart. The rat was then ventilated 60 times per min with a volume-cycled respirator. The left coronary artery was ligated as follows: a left thoracotomy incision was introduced to the rat, and the left anterior descending the coronary artery was ligated with a surgical thread, which resulted in ischemia. The treated rat was placed back to a cage and maintained. After 7 d, the treated rat was sacrificed and the blood was collected from the carotid artery for the subsequent study. The heart of the rat was also dissected for assessing the size and the degree of the myocardial infarction. The rats in the sham group experienced the same procedure except that the ligature of the left coronary artery was not performed^33^.

The dissected hearts were washed with saline and both atria were removed. The cardiac ventricles were sectioned from the apex to the base parallel to the atrioventricular groove into serial slices of 7–10 mm, which were weighed and incubated in a solution of 2,3,5-triphenyltetrazolium chloride (TTC) in PBS at 37°C for 5–10 min. This procedure stains only the healthy myocardium as red, resulting in an infarct as a pale and clearly outlined area^34^. After staining, the healthy and infarcted areas were separated by dissection, and the infarction ratio was calculated according to the following formula: infarction ratio (%) = (weight of infarcted ventricle/weight of whole ventricle) × 100.

### Langendorff perfusion and assessments

The animals used for the experiment were treated according to the protocols evaluated and approved by the ethical committee of Southeast University (Nanjing, China). Guinea pigs (250–350 g) were anesthetized with intraperitoneal pentobarbital sodium (50 mg/kg) and heparinized (1,000 U, i.p.). The heart was rapidly excised from guinea pigs and arrested in ice-cold oxygenated Locke's solution (154 mM NaCl, 5.6 mM KCl, 2.1 mM CaCl_2_, 5.9 mM NaHCO_3_, and 5.5 mM glucose 5.5 mM) before the aorta was cannulated on a Langendorff apparatus. Non-recirculating mode of retrograde perfusion with Locke's solution was carried out at a constant pressure (80 cmH_2_O) at 37°C. The isolated hearts were allowed to equilibrate for 30 min and followed by 30-min global and normothermic ischemia by halting perfusion^35^.

With three males and three females in each group, guinea pigs were randomly divided and reperfused without or with NPs for 30 min immediately after ischemia respectively. Through the mitral valve, a water-filled polyvinylchloride balloon was attached to a pressure transducer that was inserted into the left ventricular after having an incision in the left atrium. The end-diastolic pressure of the balloon inserted into the ventricle was adjusted to 25 mmHg, and the change of the pressure was recorded by a data recording system.

The LVDP and HR were measured at 5 min before ischemia, during ischemia and 10 min, 20 min, 30 min after ischemia. The values of these parameters after ischemia/reperfusion were expressed as a percentage of those obtained before ischemia.

### Hypoxia and reoxygenation

Cardiomyocytes were cultured in 96-well plate under 5% CO_2_ atmosphere at 37°C. To each confluent well, 20 μl of Fe_2_O_3_@DMSA NPs solution was added to 180 μl of cell culture medium. After 24 h, the cells, except for the normal control group, were exposed to hypoxia (95% N_2_ and 5% CO_2_) for 6 h followed by 6 h of reoxygenation (95% O_2_ and 5% CO_2_). The medium was then replaced with high sugar DMEM, and the cells were continuously cultured for additional 6 h and then subjected to biochemical and cellular analyses.

### Cell biology

To assess cytotoxicity, 20 μl MTT (5 g l^−1^) was added into each well of cell culture. After 4 h incubation at 37°C, the culture medium was removed, added with 150 μl of DMSO followed by shaking for 15 min. OD was measured with 960 automatic microplate reader at λ = 570 nm, and the value for each sample group (6 wells per group) were normalized to that of the normal group, which was set as 100%.

LDH, SOD, MDA, LD, ATP, NO and NOS in culture medium were measured by using 7550 spectrophotometer according to the manufacturer's instructions (NJBI, China). S-nitrosothiols was detected by DAN fluorometric method as described previously^36^.

To measure intracellular calcium, cells were loaded with Fluo-3/AM (5 μmol l^−1^) working solution containing 0.05% pluronic F-127 and incubated at 37°C for 45 min. After washing, the intracellular free-calcium was visualized and measured by confocal microscopy (TCS-SPII, Leica) excited at 488 nm and emitted at 530 nm. The change in Ca^2+^ was represented by changes in the percentage of relative fluorescent intensity.

### Statistical analysis

Paired Student's t-test was used to compare the data among tested groups. All the values were expressed as the mean ± SD. Results were considered statistically significant if the *p*-value was less than 0.05.

## Author Contributions

F.X., H.W., Y.L. and N.G. conceived and designed the experiments. F.X., Y.F., X.H., X.P. and S.Z. performed the experiments. L.S. and Y.Z. contributed reagents. F.X. and Y.F. wrote the paper. All authors discussed the results and commented on the manuscript.

## Supplementary Material

Supplementary Information Supplementary Information

## References

[b1] Finegold J. A., Asaria P. & Francis D. P. Mortality from ischaemic heart disease by country, region, and age: statistics from World Health Organisation and United Nations. Int. J. Cardiol. 168, 934–945 (2013). 23218570 10.1016/j.ijcard.2012.10.046 PMC3819990

[b2] Pu J. *et al.* Cardiomyocyte-expressed farnesoid-X-receptor is a novel apoptosis mediator and contributes to myocardial ischaemia/reperfusion injury. Eur. Heart J. 34, 1834–1845 (2013). 22307460 10.1093/eurheartj/ehs011 PMC3689100

[b3] Hausenloy D. J. & Yellon D. M. Myocardial ischemia-reperfusion injury: a neglected therapeutic target. J. Clin. Invest. 123, 92–100 (2013). 23281415 10.1172/JCI62874 PMC3533275

[b4] Xiong F. *et al.* Preparation, characterization of 2-deoxy-D-glucose functionalized dimercaptosuccinic acid-coated maghemite nanoparticles for targeting tumor cells. Pharm. Res. 29, 1087–1097 (2012). 22173782 10.1007/s11095-011-0653-9

[b5] Xiong F. *et al.* Rubik-like magnetic nanoassemblies as an efficient drug multifunctional carrier for cancer theranostics. J. Control Release. 172, 993–1001 (2013). 24096016 10.1016/j.jconrel.2013.09.023

[b6] Hirsch L. R. *et al.* Nanoshell-mediated near-infrared thermal therapy of tumors under magnetic resonance guidance. Proc. Natl Acad. Sci. USA 100, 13549–13554 (2003). 14597719 10.1073/pnas.2232479100 PMC263851

[b7] Bergemann C., Muller-Schulte D. & Oster J. Magnetic ion-exchange nano- and microparticles for medical, biochemical and molecular biological applications. Magn. Magn. Mater. 194, 45–52 (1999).

[b8] Gao L. *et al.* Intrinsic peroxidase-like activity if ferromagnetic nanoparticles. Nature Nanotech 2, 577–583 (2007). 10.1038/nnano.2007.260 18654371

[b9] Chen Z. *et al.* Dual enzyme-like activities of iron oxide nanoparticles and their implication for diminishing cytotoxicity. ACS Nano. 6, 4001–4012 (2012). 22533614 10.1021/nn300291r

[b10] Zhou R. *et al.* Cardioprotective effect of water and ethanol extract of Salvia miltiorrhiza in an experimental model of myocardial infarction. J. Ethnopharmacol. 139, 440–446 (2012). 22138518 10.1016/j.jep.2011.11.030

[b11] Chang P. N. *et al.* Analysis of cardioprotective effects using purified Salvia miltiorrhiza extract on isolated rat hearts. J. Pharmacol. Sci. 101, 245–9 (2006). 16837771 10.1254/jphs.fpj05034x

[b12] Wu T., Ni J. & Wu J. Danshen (Chinese medicinal herb) preparations for acute myocardial infarction. Cochrane Database Syst. Rev. 16, CD004465 (2008). 18425903 10.1002/14651858.CD004465.pub2 PMC8406986

[b13] Asati A., Santra S., Kaittanis C. & Perez J. M. Surface-charge-dependent cell localization and cytotoxicity of cerium oxide nanoparticles. ACS Nano, 4, 5321–5331 (2010). 20690607 10.1021/nn100816s PMC2947560

[b14] Hirsch V. *et al.* Surface charge of polymer coated SPIONs influences the serum protein adsorption, colloidal stability and subsequent cell interaction in vitro. Nanoscale 5, 3723–3732 (2013). 23334060 10.1039/c2nr33134a

[b15] Fröhlich E. The role of surface charge in cellular uptake and cytotoxicity of medical nanoparticle. Int J Nanomedicine 7, 5577–5591 (2012). 23144561 10.2147/IJN.S36111 PMC3493258

[b16] Demortière A. *et al.* Size-dependent properties of magnetic iron oxide nanocrystals. Nanoscale 3, 225–332 (2011). 21060937 10.1039/c0nr00521e

[b17] Chatterjee J., Haik Y. & Chen C. J. Size dependent magnetic properties of iron oxide nanoparticles. J Magn Magn Mater. 257, 113–118 (2003).

[b18] Wang Q. D., Pernow J., Sjöquist P. O. & Rydén L. Pharmacological possibilities for protection against myocardial reperfusion injury. Cardiovasc. Res. 55, 25–37 (2002). 12062706 10.1016/s0008-6363(02)00261-4

[b19] Sokhanenkova A. E., Sokhanenkov Miu., Afanas'eva Eiu. & Arzamastsev E. V. Characteristics of pharmacological and toxic effects of verapamil during cardiac arrhythmia in thyrotoxic and hypothyroid rats. Kardiologiia 48, 57–61 (2008). 18729838

[b20] Bechtel L. K., Haverstick D. M. & Holstege C. P. Verapamil toxicity dysregulates the phosphatidylinositol 3-kinase pathway. Acad. Emerg. Med. 15, 368–374 (2008). 18370992 10.1111/j.1553-2712.2008.00088.x

[b21] Wang L. D. & Nang B. S. The toxicity of Verapamil to cardiomyocytes. Chin. J. Clin. Pharmacol. Ther. 2, 178–179 (2000).

[b22] Gawryszewski V. P. & Souza Mde. F. Mortality due to cardiovascular diseases in the Americas by region, 2000–2009. Sao Paulo Med J. 132, 105–10 (2014). 24714991 10.1590/1516-3180.2014.1322604 PMC10896582

[b23] Hausenloy D. J. & Yellon D. M. Myocardial ischemia-reperfusion injury: a neglected therapeutic target. J Clin Invest. 123, 92–100 (2013). 23281415 10.1172/JCI62874 PMC3533275

[b24] Wang Q. D., Morcos E., Wiklund P. & Pernow J. L-arginine enhances functional recovery and Ca(2+)-dependent nitric oxide synthase activity after ischemia and reperfusion in the rat heart. J Cardiovasc Pharmacol. 29, 291–296 (1997). 9057081 10.1097/00005344-199702000-00020

[b25] Minhas K. M. *et al.* Xanthine oxidoreductase inhibition causes reverse remodeling in rats with dilated cardiomyopathy. Circ Res. 98, 271–279 (2006). 16357304 10.1161/01.RES.0000200181.59551.71

[b26] Johnson G. R., Tsao P. S., Mulloy D. & Lefer A. M. Cardioprotective effects of acidified sodium nitrite in myocardial ischemia with reperfusion. J Pharmacol Exp Ther. 252, 35–41 (1990). 2153807

[b27] Williams M. W., Taft C. S., Ramnauth S., Zhao Z. Q. & Vinten-Johansen J. Endogenous nitric oxide (NO) protects against ischaemia-reperfusion injury in the rabbit. Cardiovasc Res. 30, 79–86 (1995). 7553727

[b28] Prime T. A. *et al.* A mitochondria-targeted S-nitrosothiol modulates respiration, nitrosates thiols, and protects against ischemia-reperfusion injury. Proc Natl Acad Sci U S A 106, 10764–10769 (2009). 19528654 10.1073/pnas.0903250106 PMC2696550

[b29] Poteser M., Romanin C., Schreibmayer W., Mayer B. & Groschner K. S-nitrosation controls gating and conductance of the alpha 1 subunit of class C L-type Ca(2+) channels. J Biol Chem. 276, 14797–14803 (2001). 11278396 10.1074/jbc.M008244200

[b30] Gao L., Giglio K. M., Nelson J. L., Sondermann H. & Travis A. J. Ferromagnetic nanoparticles with peroxidase-like activity enhance the cleavage of biological macromolecules for biofilm elimination. Nanoscale 6, 2588–2593 (2014). 24468900 10.1039/c3nr05422e PMC3951791

[b31] Huang G. *et al.* Superparamagnetic iron oxide nanoparticles: amplifying ROS stress to improve anticancer drug efficacy. Theranostics 3, 116–126 (2013). 23423156 10.7150/thno.5411 PMC3575592

[b32] Zhang S. *et al.* The effect of iron oxide magnetic nanoparticles on smooth muscle cells. Nanoscale Res. Lett. 4, 70–77 (2009).

[b33] Guo Q. *et al.* Protective effect of corocinnarine on ischemia-reperfusion myocardium of rats and its relation to anti-lipid peroxidation. Chin. Heart J. 14, 4–6 (2002).

[b34] Hopkins T. A., Ouchi N., Shibata R. & Walsh K. Adiponectin actions in the cardiovascular system. Cardiovasc. Res. 74, 11–18 (2007). 17140553 10.1016/j.cardiores.2006.10.009 PMC1858678

[b35] Skrzypiec-Spring M., Grotthus B., Szelag A. & Schulz R. Isolated heart perfusion according to Langendorff—still viable in the new millennium. J. Pharmacol. Toxicol. Methods 55, 113–126 (2007). 16844390 10.1016/j.vascn.2006.05.006

[b36] Park J. K. & Kostka P. Fluorometric detection of biological S-nitrosothiols. Anal. Biochem. 249, 61–66 (1997). 9193709 10.1006/abio.1997.2159

