# Supplementary material for: Cardioprotective activity of iron oxide nanoparticles
Source: Sci Rep. 2015 Feb 26;5:8579. doi: 10.1038/srep08579 (PMC4341209; doi:10.1038/srep08579)
Supplement: Supplementary Information [file srep08579-s1.pdf]

Cardioprotective activity of iron oxide nanoparticles

Fei Xiong, Hao Wang, Yidong Feng, Yunman Li, Xiaoqing Hua, Xingyun Pang, Song Zhang, Lina Song, Yu Zhang and Ning Gu *

*email: guning@seu.edu.cn

Contents

Page 2-4. Materials and Methods

Page 5. Figure S1: The average hydrodynamic diameter of 9.8 nm Fe_2O_3@DMSA NPs (84.8 nm), 10.1 nm Fe_2O_3@APTs NPs (79.0 nm), 9.6 nm Fe_2O_3@Glu NPs (71.1 nm) and 35.2 nm Fe_2O_3@DMSA NPs (362.5 nm).

Page 5. Figure S2: TEM images of Fe_2O_3@ATPs NPs (10.1 nm) and Fe_2O_3@ATPs NPs (9.6 nm).

Page 6. Figure S3: The Langendorff hearts perfused with 0.1 mg ml^{-1} of Verapamil and 0.001mg ml^{-1} of *Salvia miltiorrhiza* extract in Locke's solution after 30 min of ischemia.

Materials

2, 3-dimercaptosuccinic acid, 3-amino-propyltriethoxysilane and L-glutamic acid were purchased from Alpha Chem Co. Ltd., Sigma Ltd. and Shanghai Xinghui Co., Ltd, respectively. Verapamil Hydrochloride Injection, produced by Shanghai Wellhope Pharmaceutical Co., Ltd. Lot Number: 111001. It should be prepared to solution with sterilized ultrapure water. Salvia injection (*Salvia miltiorrhiza* extract, Lyophilized Powder), produced by Harbin Pharmaceutical Group Chinese Medicine Plant Two, Lot Number: 110152. It should be prepared to solution with ultrapure water.

The animals used for the experiment were treated according to the protocols evaluated and approved by the ethical committee of Southeast University (Nanjing, China). Guinea pigs: weight from 250-350g, half male and half female, were provided by Nanjing Jiangning District Qinglongshan animal breeding farms. Experiments in vivo were performed on male Sprague-Dawley rats (200-250 g). Rats were purchased from Shanghai Laboratory Animal Center of the Chinese Academy of Sciences. Rats were allowed one week to adapt themselves to the environment before being used for experiments. All the animals were maintained on a 12 h light/12 h dark cycle, kept in a temperature-controlled room (about 22-26 °C).

Methods

Synthesis of IONPs

Fe_2O_3 NPs with approximately 10 nm were synthesized by chemical co-precipitation and subsequently stabilized with DMSA, APTs and Glu. In detail, a solution of

$FeCl_3 \cdot 6H_2O$ (0.01 M) and $FeSO_4 \cdot 7H_2O$ (0.006 M) was prepared under a stream of N_2 protecting. The aqueous ammonia solution (1.5 M) was added into the mixed solution with violently stirring until the pH of the solution was raised to 9. The obtained Fe_3O_4 NPs were washed with water and ethanol for 2-5 times by magnetic separation. The pH of magnetite NPs was adjusted to 3.0 using 0.1 M HCl. Then theses Fe_3O_4 NPs were oxidized into reddish-brown Fe_2O_3 NPs by air for 1 h at 95-100 °C.

Subsequently, the Fe_2O_3 NPs were coated with DMSA, APTs and Glu according to the process described elsewhere. Finally, the products were washed with water.

35.2 nm Fe_2O_3 NPs were purchased from XCNM Co., Ltd (China) and coated with DMSA as the same method mentioned above.

Characterization of IONPs

The core diameters of NPs were characterized by TEM (JEM-200CX, JEOL). A drop of NPs suspension was placed on a carbon-coated 300 mesh copper grid. Then the sample was dried at room temperature before it was attached to the holder.

The hydrodynamic diameters and zeta potential were measured at 25 °C by electrophoresis instrument (Brookhaven Zetaplus, Malvern). All samples were diluted 100 times by water.

Cell culture

The abdominal and chest skins of 1- to 3-day-old neonatal SD rats were sterilized with 2% iodine tincture and 75% ethanol respectively, chest skin was cut off with irisscissors to

expose hypodermis. Then sterilizing hypodermis with 2% iodine tincture and 75% ethanol, operator took out the heart after opening breast, washed out residue hematocele in bottle dishes with PBS, isolated ventricles and minced them into 1mm^3 fragments to get ready for digesting.

Cardiomyocytes suspension was obtained through sequential digestion. For selective enrichment of cardiomyocytes, selective plating technique triangle was chosen. Dissociated cell suspension was preplated for 180 min, 37°C in flasks or plates, during which the epithelial cells attached quickly to the bottom of the culture plates; non-adherent cells, mostly myocytes, were seeded to a culture 96-well plate with at a density of 5×10^5 ml$^{-1.}$ Cells were grown in DMEM in the presence of calf serum under a 5% CO_2 atmosphere in air saturated with water vapor at 37°C. Culture medium was renewed every 2 days according adherence rates of cells and pH of medium.

Cellular uptake of IONPs

For Prussian blue staining, the cells were fixed with 2.5% glutaraldehyde at 4 °C for 1 h, washed, and incubated for 30 minutes with mixed solution of 4% potassium ferric-ferrocyanide and 4% hydrochloric acid. After being washed, the cells were evaluated for iron staining using light microscopy (XDS-1, COI, China).

For Fe concentration measurement, the cell layer was dissolved in 30% v/v HCl. 1ml of 6 % potassium ferrocyanide was then added and the absorbance was read after 10 min at 690 nm. A standard curve of ferric chloride solution was recorded in the same conditions to calculate the cellular uptake of iron.

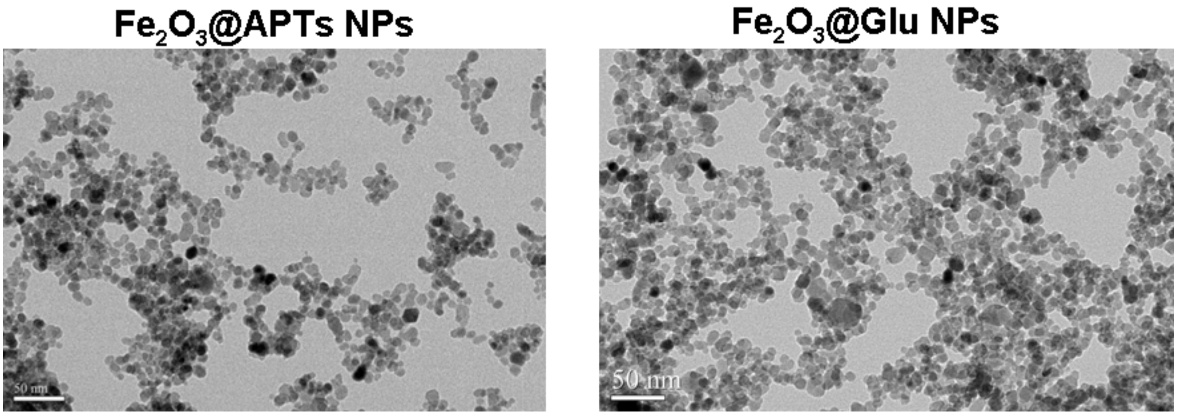

Supplementary Figure 1. TEM images of Fe_2O_3@ATPs NPs (10.1 nm) and Fe_2O_3@ATPs NPs (9.6 nm).

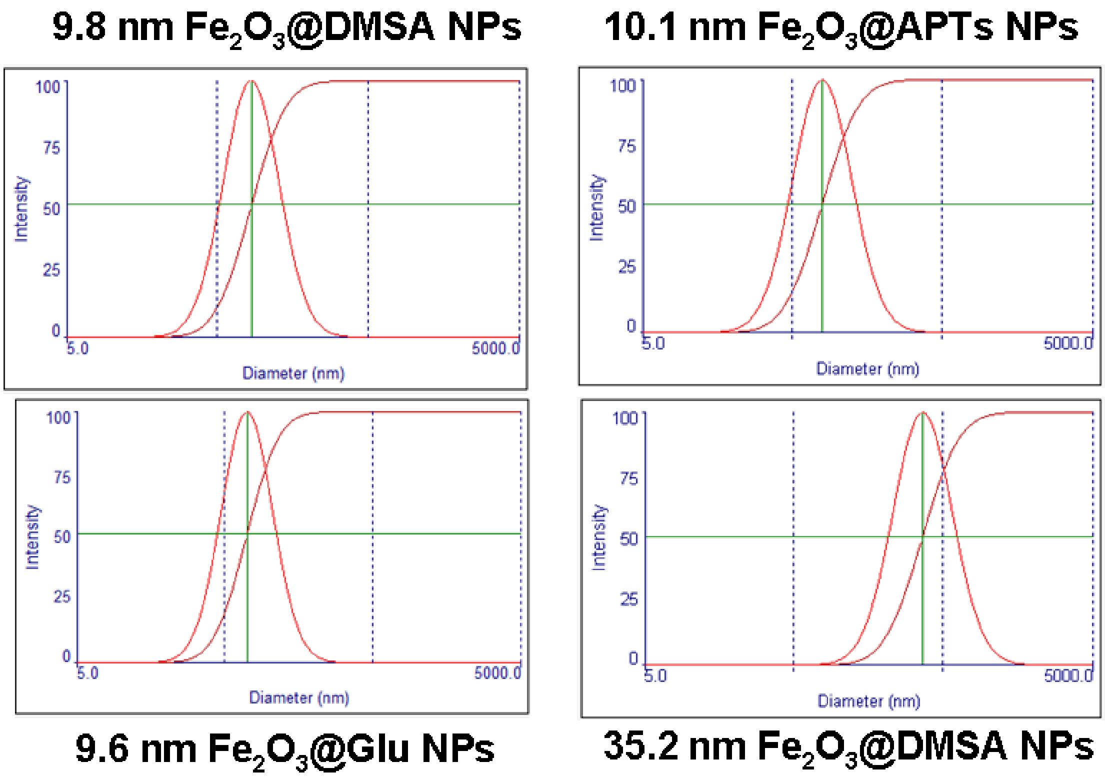

Supplementary Figure 2. The average hydrodynamic diameter of 9.8 nm Fe_2O_3@DMSA NPs (84.8 nm), 10.1 nm Fe_2O_3@APTs NPs (79.0 nm), 9.6 nm Fe_2O_3@Glu NPs (71.1 nm) and 35.2 nm Fe_2O_3@DMSA NPs (362.5 nm).

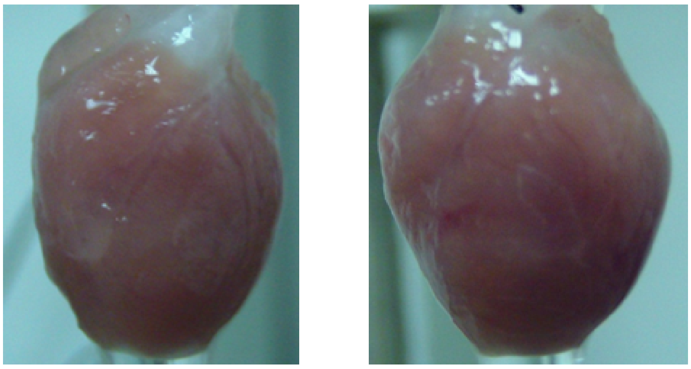

Supplementary Figure 3. The Langendorff hearts perfused with 0.1 mg ml^{-1} of Verapamil and 0.001mg ml^{-1} of *Salvia miltiorrhiza* extract in Locke’s solution after 30 min of ischemia.
